# Enterococcus species: insights into antimicrobial resistance and whole-genome features of isolates recovered from livestock and raw meat in Ghana

**DOI:** 10.3389/fmicb.2023.1254896

**Published:** 2023-12-05

**Authors:** Grebstad Rabbi Amuasi, Esther Dsani, Christian Owusu-Nyantakyi, Felicia A. Owusu, Quaneeta Mohktar, Pernille Nilsson, Bright Adu, Rene S. Hendriksen, Beverly Egyir

**Affiliations:** ^1^Department of Bacteriology, Noguchi Memorial Institute for Medical Research, College of Health Sciences, University of Ghana, Accra, Ghana; ^2^Veterinary Services Department, Ministry of Food and Agriculture, Accra, Ghana; ^3^Department of Immunology, Noguchi Memorial Institute for Medical Research, College of Health Sciences, University of Ghana, Accra, Ghana; ^4^National Food Institute, Research Group for Global Capacity Building, WHO Collaborating Centre for Antimicrobial Resistance in Foodborne Pathogens and Genomics, FAO Reference Laboratory for Antimicrobial Resistance, European Union Reference Laboratory for Antimicrobial Resistance, Technical University of Denmark, Kongens Lyngby, Denmark

**Keywords:** Enterococcus spp., antimicrobial resistance, whole-genome sequencing, livestock, raw meat, Africa

## Abstract

**Introduction:**

*Enterococcus* spp. have gradually evolved from commensals to causing life-threatening hospital-acquired infections globally due to their inherent antimicrobial resistance ability and virulence potential. *Enterococcus* spp. recovered from livestock and raw meat samples were characterized using antimicrobial susceptibility testing and whole-genome sequencing.

**Materials and methods:**

Isolates were confirmed using the MALDI-ToF mass spectrometer, and antimicrobial susceptibility was determined using the Kirby-Bauer disk diffusion method. Whole genome sequencing was performed on isolates resistant to two or more antibiotics. Bioinformatics analysis was performed to determine sequence types, resistance and virulence gene content and evolutionary relationships between isolates from meat and livestock samples, and other enterococci genomes curated by PATRIC. eBURST analysis was used to assign genomes to clonal complexes.

**Results:**

*Enterococcus* spp. were predominantly *E. faecalis* (96/236; 41%) and *E. faecium* (89/236; 38%). Overall, isolates showed resistance to erythromycin (78/236; 33%), tetracycline (71/236; 30%), ciprofloxacin (20/236; 8%), chloramphenicol (12/236; 5%), linezolid (7/236; 3%), ampicillin (4/236; 2%) and vancomycin (1/236, 0.4%). Resistance to two or more antimicrobial agents was detected among 17% (*n* = 40) *Enterococcus* spp. Resistance genes for streptogramins [*lsa(A), lsa(E), msr(C)*], aminoglycosides [*aac(6′)-Ii, aph(3′)-III, ant(6)-Ia, aac(6′)-aph(2″), str],* amphenicol [*cat*], macrolides [*erm(B), erm(T), msr(C)*], tetracyclines [*tet(M), tet(L), tet(S)*] and lincosamides [*lsa(A), lsa(E), lnu(B)*] were detected among the isolates. Genes for biofilm formation, adhesins, sex pheromones, cytolysins, hyaluronidase, oxidative stress resistance, quorum-sensing and anti-phagocytic activity were also identified. Potential plasmids with replicon sequences (*rep1, rep2, repUS43, repUS47, rep9a, rep9b*) and other mobile genetic elements (*Tn917, cn_5536_ISEnfa1, Tn6009, ISEnfa1, ISEfa10*) were detected. Clinically relevant *E. faecium* ST32 and ST416 clones were identified in meat samples.

**Conclusion:**

The occurrence of antimicrobial-resistant *Enterococcus* spp. in livestock and raw meat samples, carrying multiple resistance and virulence genes, including known clones associated with hospital-acquired infections, underscores the critical need for employing robust tools like whole genome sequencing. Such tools provide detailed data essential for ongoing surveillance efforts aimed at addressing the challenge of antimicrobial resistance with a focus on one health.

## 1 Introduction

*Enterococcus* spp. exist as commensals in the gut of warm-blooded animals and humans but possess virulence genes, and are recognized as opportunistic pathogens that can cause a variety of hospital-acquired infections, such as urinary tract and intra-abdominal infections, bacteremia, and endocarditis (Iweriebor et al., 2015; Ngbede et al., 2017). *E. faecium* and *E. faecalis* are commonly associated with opportunistic infections, with other species being rarely pathogenic (Beukers et al., 2017). Enterococci have notable resistance to adverse environmental conditions, hence the capacity to colonize different ecological niches and spread within the food chain through contaminated animals and foods (Klibi et al., 2013). The inherent high antimicrobial resistance (AMR) ability of *Enterococcus* spp., and their ability to genetically acquire and transmit antimicrobial drug resistant determinants among themselves and other bacteria in the environment, presents a significant challenge for therapeutic measures (Iweriebor et al., 2015).

One common phenomenon that increases AMR among bacterial species including Enterococci is the use of antibiotics in animal production, which subsequently creates ideal conditions for evolution and selection of resistant strains (Iweriebor et al., 2015; Ngbede et al., 2017). Several studies have documented the widespread use of antibiotics in animal husbandry in Ghana, with majority of farmers using antibiotics for the purposes of treatment or prophylaxis, and at a lesser extent for growth stimulation (Donkor et al., 2012; Osei Sekyere, 2014; Boamah et al., 2016). Although Donkor et al. (2012) reports that there is no significant difference in the frequency of antimicrobial usage among different animals (cattle, goat, sheep, pig, poultry), Osei Sekyere (2014) indicates that the frequency of antimicrobial usage is influenced by the size or financial status of farms and mixed farming practices. In an effort to reduce costs and maintain profits, farmers in Ghana disregard the recommended waiting period after administering antibiotics to food animals by selling animal products like eggs and meat for human consumption (Boamah et al., 2016; Nkansa, 2020). This causes persistence of antibiotic residues in animal products, thereby exposing microorganisms in animals and humans to sub-inhibitory concentrations of antibiotic, leading to the emergence and spread of antibiotic-resistant bacterial strains through food or other environmental pathways (Iweriebor et al., 2015; Boamah et al., 2016; McEwen and Collignon, 2018; Collignon and McEwen, 2019). Unavailability of regulations regarding the use of antimicrobials in animals, limited involvement of veterinarians in the administration of antibiotics, and the unavailability of optimal storage conditions for antibiotics are among factors identified to be influencing the extensive and inappropriate use of antimicrobials in animal husbandry in the country (Donkor et al., 2012; Osei Sekyere, 2014; Boamah et al., 2016; Nkansa, 2020).

Enterococci are one of two indicator bacteria used to study the extent of AMR in populations owing to their proven resistance and virulence transmissibility as demonstrated in surveillance programs implemented in developed countries (Bager, 2000; European Food Safety Authority, European Centre for Disease Prevention and Control, 2013). Although the multidrug resistance potential of *Enterococcus* spp. has been demonstrated in other studies across Africa (Klibi et al., 2013; Iweriebor et al., 2015; Ngbede et al., 2017; Katakweba et al., 2018), data on *Enterococci* of human, animal and environmental origin in Ghana is scarce. Reports (Obiri-Danso et al., 2003, 2005; Ayum and Gifty, 2010; Labi et al., 2015; Quansah et al., 2018, 2020; Akrong et al., 2019; Akita et al., 2021) of isolated *Enterococcus* spp. from human clinical sources and environmental sources in Ghana have consistently lacked genomic characterization. Given that there are clear reports of inappropriate use of antibiotics on farms in Ghana, the limited application of genomic characterization obscures understanding of the genetic basis of ensuing antibiotic resistance, including the evolution and transmission of resistance genes, and the dynamics of resistance in bacterial populations. The application of whole genome sequencing (WGS) offers extensive genomic information and is valuable for characterizing bacterial isolates and conducting genomic surveillance of AMR in bacteria. This study employed WGS and antimicrobial susceptibility testing to examine *Enterococcus* spp. recovered from archived raw meat and livestock samples collected in Ghana.

## 2 Materials and methods

### 2.1 Study site and isolates

Samples used in the study were obtained from three livestock farms and three slaughterhouses in southern Ghana independent of each other, from 2018 to 2019 and archived at the Bacteriology Department, Noguchi Memorial Institute for Medical Research. Livestock samples consisted of fecal swabs collected from cattle, goats, pigs, poultry and sheep. Meat samples were collected from the thigh, brisket and flank/mid-loin of carcasses at slaughterhouses using sterile swabs and stored in brain heart infusion broth. Carcasses sampled included beef, chevon and mutton. One milliliter of each archived broth sample was pre-enriched with 9 mL of trypticase soy broth and incubated overnight at 37°C. A loopful of pre-enriched samples was then cultured on Bile Esculin Azide agar (Merck, Germany) and incubated for 18–24 h at 37°C. Presumptive *Enterococcus* spp. were seen as small transparent colonies with black halos. Presumptive *Enterococcus* spp. were confirmed using MALDI-TOF mass spectrometer (Bruker, Billerica, MA, USA) following subculture on nutrient agar (Oxoid, Basingstoke, Hants, United Kingdom).

Antimicrobial susceptibility was determined using the Kirby-Bauer disk diffusion method and interpreted with reference to the Clinical and Laboratory Standards Institute (CLSI) guideline (CLSI, 2021). Antibiotics used included ampicillin (10ug), vancomycin (30ug), erythromycin (15ug), tetracycline (30ug), ciprofloxacin (5ug), chloramphenicol (30ug) and linezolid (30ug) from Oxoid (Basingstoke, Hants, UK). The reference strain *S. aureus* ATCC 25923 was applied as quality control.

### 2.2 Whole-genome sequencing and analysis

Genomic DNA was extracted from *Enterococcus* spp. resistant to two or more antimicrobial agents using QIAamp^®^ DNA mini kit (QIAGEN Inc. GmbH, Holden, Germany), with reference to the manufacturer’s protocol. Libraries of DNA were prepared using the Illumina^®^ DNA Prep (M) Tagmentation kit (Illumina Inc. San Diego, CA 92122, United States) followed by multiplexing and 300 bp paired-end sequencing on the Illumina Miseq platform (Illumina, San Diego, CA, United States). Fastq files generated were assessed for quality using Fastqc (v0.11.9) and trimmed with Trimmomatic (v.0.39) at a minimum quality threshold of ≥ Q20 (Andrews, 2010; Bolger et al., 2014). Reads that met the quality threshold (Q20 or higher) after trimming, were subjected to de-novo assembly using Unicycler (v0.4.8). De-novo assembly was performed using the following parameters: minimum contig size of 200 bp, number of contigs <400, and minimum coverage of 20x. All assembled sequence data were submitted to GenBank and assigned accession numbers under Bio project PRJNA851427.

Assembled genomes were analyzed using online tools hosted by Center for Genomic Epidemiology.^1^ KmerFinder (v3.2; Hasman et al., 2014) was applied to confirm the identity of isolates sequenced. Kraken (v2.1.3; Wood et al., 2019) and Bracken (v2.6.1; Lu et al., 2017) were used to assess contamination. Resistance determinants were predicted using ResFinder (v4.1; Bortolaia et al., 2020), virulence genes by VirulenceFinder (v2.0; Joensen et al., 2014), plasmids and other mobile genetic elements by PlasmidFinder (v2.1; Carattoli et al., 2014) and MobileElementFinder (v1.0.3; Johansson et al., 2021) respectively. Sequence types were determined using the MLST option (v2.0.9; Larsen et al., 2012); eBURST analysis from PubMLST (https://pubmlst.org/bigsdb?db=pubmlst_efaecalis_seqdef&page=plugin&name=BURST&scheme_id=1) was used to assign genomes to clonal complexes based on their MLST profiles (Feil et al., 2004; Leavis et al., 2006; Jolley et al., 2018).

Using the CSI phylogeny tool, evolutionary relationships between *E. faecalis* and *E. faecium* isolates from this study and other African Enterococci genomes (curated by Bacterial and Viral Bioinformatics Resource Center),^2^ were analyzed and inferred from single nucleotide polymorphisms (SNPs), by comparing assembled sequences to the reference genomes *E. faecium* SRR24 (NZ_CP038996.1) and *E. faecalis* V583 (226185.9). Close genetic relatedness was determined based on a pairwise SNP difference below 10 between the genomes (Weterings et al., 2015). The maximum-likelihood phylogenetic tree generated was annotated in Interactive Tree of Life (iTOL; Letunic and Bork, 2021). The accession numbers of downloaded genomes have been included in the Supplementary materials.

### 2.3 Statistical analysis

Statistical analysis was performed to assess the difference in *Enterococcus* spp. isolation from the respective samples and to also assess the correlation between resistance phenotypes and their respective genotype using the chi square test of independence and logistic regression. A significance level of α = 0.05 or lower was employed to determine statistical significance. Analyses were carried out using Stata version 16.

## 3 Results

### 3.1 Distribution of *Enterococcus* species recovered

In total, the 374 samples tested originated from raw meat (*n* = 200) and livestock (*n* = 174). Meat samples included chevon (*n* = 105), beef (*n* = 80) and mutton (*n* = 15) whereas livestock samples comprised of sheep (*n* = 50), pigs (*n* = 40), cattle (*n* = 39), goats (*n* = 35) and poultry (*n* = 10; Table 1). Out of the 374 samples, 236 (63%) *Enterococcus* spp. were recovered, with *E. faecalis* (*n* = 96; 41%) and *E. faecium* (*n* = 89; 38%) predominating. *E. hirae*, *E. casseliflavus*, E*. galinarum*, *E. thailandicus*, among others were also isolated in this study (Table 1). The isolation of *Enterococcus* spp. from the different samples was not statistically significant (Table 1).

**Table 1:** Distribution of *Enterococcus* spp. recovered.

		Meat (*n* = 200)							
Samples	Total	*E. faecalis*	*E. faecium*	*E. hirae*	*E. casseliflavus*	*E. galinarum*	*E. thailandicus*	*E. durans*	*p*-value
Chevon (*n* = 105)	37	4	28	1	1	–	–	2	
Beef (n = 80)	28	5	17	3	1	2	–	–	
Mutton (*n* = 15)	2	1	–	1	–	–	–	–	0.256
		Livestock (*n* = 174)							
Sheep (*n* = 50)	50	30	15	1	1	1	–	–	
Pig (*n* = 40)	38	14	12	2	4	1	4	1	
Cattle (*n* = 39)	37	16	10	3	5	1	–	–	
Goat (*n* = 35)	34	19	6	4	–	2	1	–	
Poultry (*n* = 10)	10	7	1	–	–	2	–	–	0.060

Other *Enterococcus* spp. isolated included: *E. avium* [Sheep (*n* = 2), Cattle (*n* = 1)], *E. pseudoavium* [Meat (*n* = 1), Cattle (*n* = 1)], *E. innesii* [Goat (*n* = 1)], E. lactis [Goat (*n* = 1)].

### 3.2 Antimicrobial susceptibility profile of *Enterococcus* species

*Enterococcus* spp. recovered were resistant to erythromycin (78/236; 33%), tetracycline (71/236; 30%), ciprofloxacin (20/236; 8%), chloramphenicol (12/236; 5%), linezolid (7/236;3%), ampicillin (4/236; 2%) and vancomycin (1/236; 0.4%; Table 2). Resistance to two or more antimicrobial agents was detected among 17% (*n* = 40) *Enterococcus* spp., with *E. faecium* and *E. faecalis* accounting for 98% (39/40) of this population. These isolates were resistant to erythromycin (38/40; 95%), tetracycline (39/40; 98%), ciprofloxacin (10/40; 25%), chloramphenicol (8/40; 20%), linezolid (1/40; 2.5%) and ampicillin (1/40; 2.5%). Use of tetracycline (2/3; 67%), penicillin (2/3; 67%) and colistin (1/3; 33%) was recorded in the livestock farms sampled.

**Table 2:** Antimicrobial resistance profile of *Enterococcus* spp. recovered from livestock and raw meat samples.

Antibiotic	*E. faecalis (n = 96)*	*E. faecium (n = 89)*	*E. hirae (n = 15)*	*E. galinarum (n = 9)*	*E. thailandicus (n = 5)*	*E. durans (n = 3)*	*E. pseudoavium (n = 2)*	*E. innesii (n = 1)*	*E. lactis (n = 1)*
Ampicillin	*3(3)*	*–*	*1(7)*	*–*	*–*	*–*	*–*	*–*	*–*
Chloramphenicol	*8(8)*	*3(3)*	*1(7)*	*–*	*–*	*–*	*–*	*–*	*–*
Ciprofloxacin	*10(10)*	*7(8)*	*1(7)*	*1(11)*	*–*	*–*	*–*	*1(100)*	*–*
Erythromycin	*33(34)*	*32(36)*	*2(13)*	*3(33)*	*5(100)*	*–*	*1(50)*	*–*	*1(100)*
Linezolid	*5(5)*	*2(2)*	*–*	*–*	*–*	*–*	*–*	*–*	*–*
Tetracycline	*39(41)*	*21(24)*	*3(20)*	*4(44)*	*–*	*2(67)*	*–*	*1(100)*	*1(100)*
Vancomycin	*–*	*–*	*–*	*1(11)*	*–*	*–*	*–*	*–*	*–*

Data is presented as frequency and percentages in parentheses. *E. casseliflavus* (*n* = 12) and *E. avium* (*n* = 3) were not resistant to any of the antibiotics tested.

### 3.3 Genomic characteristics of *Enterococcus* species

A summary of the genome characteristics of *Enterococcus* spp. sequenced is presented in Table 3. All assembled genomes passed QC with Q scores >20, minimum coverage of 20, number of contigs <400 and minimum contig size of 200 bp. The assembled genomes ranged in size from 2.5–3.1 Mb, with average GC content of 37.6.

**Table 3:** Genome characteristics of sequenced *Enterococcus* spp.

Isolate ID	Genome accession	Species	Source	Genome size	Number of contigs	GC content (%)	Longest contig size (bp)	N50 value (bp)	L50 value	Coverage
2	JAMXFQ000000000	*Enterococcus faecium*	Pig	2,768,647	60	37.8	611,566	255,931	4	30
3	JAMXGQ000000000	*Enterococcus faecium*	Pig	2,546,321	217	38.1	124,571	42,625	20	89
18F	JAMXFU000000000	*Enterococcus faecalis*	Pig	2,783,545	68	37.4	264,324	136,395	8	25
20F	JAMXGB000000000	*Enterococcus faecium*	Pig	2,690,886	34	38.1	436,979	158,269	5	25
32F	JAMXHO000000000	*Enterococcus faecalis*	Cattle	2,858,470	55	37.4	542,786	235,922	5	91
38F	JAMXHG000000000	*Enterococcus faecalis*	Cattle	2,904,169	76	37.4	545,589	235,913	5	69
30M	JAMXFZ000000000	*Enterococcus faecium*	Cattle	2,641,064	78	38.2	219,791	106,068	9	72
36M	JAMXGA000000000	*Enterococcus faecium*	Cattle	2,612,814	43	38.1	291,859	110,559	8	23
76F	JAMXGW000000000	*Enterococcus faecalis*	Sheep	3,018,758	46	37.3	818,026	269,475	3	72
78M	JAMXFR000000000	*Enterococcus faecalis*	Sheep	3,053,743	54	37.3	471,758	197,063	5	35
85	JAMXHL000000000	*Enterococcus faecalis*	Sheep	2,971,150	115	37.3	227,276	121,098	10	91
47F	JAMXGF000000000	*Enterococcus faecalis*	Sheep	2,960,990	30	37.2	1,333,726	307,737	2	35
78	JAMXGD000000000	*Enterococcus faecium*	Sheep	2,897,022	73	38	374,155	150,789	7	113
81	JAMXGE000000000	*Enterococcus faecalis*	Sheep	2,867,305	28	37.4	878,189	592,527	2	90
72F	JAMXHK000000000	*Enterococcus faecalis*	Sheep	2,854,388	34	37.4	1,361,034	592,527	2	85
73	JAMXHC000000000	*Enterococcus faecalis*	Sheep	2,960,916	31	37.2	1,333,726	307,737	2	82
74	JAMXFN000000000	*Enterococcus faecalis*	Sheep	2,865,094	34	37.4	1,361,034	592,527	2	70
75	JAMXGJ000000000	*Enterococcus faecalis*	Sheep	2,790,259	97	37	278,556	177,634	7	59
79F	JAMXGX000000000	*Enterococcus faecium*	Sheep	2,786,873	70	38	280,013	197,863	6	69
82	JAMXHH000000000	*Enterococcus faecalis*	Sheep	2,861,305	33	37.4	814,177	361,734	3	34
83	JAMXHI000000000	*Enterococcus faecium*	Sheep	2,841,986	98	38.1	370,335	195,765	6	49
55F	JAMXGO000000000	*Enterococcus faecalis*	Goat	3,056,778	71	37.2	408,134	218,945	5	58
65F	JAMXFO000000000	*Enterococcus faecalis*	Goat	3,055,606	68	37.2	408,134	183,061	6	33
65	JAMXFP000000000	*Enterococcus faecalis*	Goat	2,746,881	39	37.5	541,948	220,283	4	29
67	JAMXHJ000000000	*Enterococcus faecium*	Goat	3,891,554	383	38.9	168,836	31,022	31	41
70	JAMXHD000000000	*Enterococcus faecalis*	Goat	2,910,012	34	37.4	909,064	361,272	3	62
71	JAMXHB000000000	*Enterococcus faecalis*	Goat	2,965,321	41	37.4	909,064	361,734	3	70
67 M	JAMXFY000000000	*Enterococcus faecium*	Goat	2,695,895	70	38.2	371,764	163,672	6	50
68	JAMXGG000000000	*Enterococcus lactis*	Goat	2,648,130	77	38.2	362,111	144,863	6	52
88 L	JAMXGV000000000	*Enterococcus faecalis*	Poultry	3,041,893	63	37.2	825,893	264,324	3	67
90	JAMXGP000000000	*Enterococcus faecalis*	Poultry	3,096,512	76	37.1	800,089	301,301	3	90
91	JAMXFV000000000	*Enterococcus faecalis*	Poultry	2,944,796	61	37.3	825,893	264,386	3	66
104	JAMXGT000000000	*Enterococcus faecalis*	Poultry	3,042,023	63	37.2	825,893	264,379	3	92
89	JAMXGU000000000	*Enterococcus faecalis*	Poultry	2,854,080	34	37.3	968,755	292,445	3	83
90e	JAMXGI000000000	*Enterococcus faecium*	Meat	2,933,704	213	37.9	173,661	72,156	13	52
35E	JAMXFX000000000	*Enterococcus faecium*	Meat	2,474,444	47	38	272,577	174,454	6	40
70	JAMXFW000000000	*Enterococcus faecium*	Meat	2,841,712	97	38.1	370,335	195,765	6	34
75	JAMXGJ000000000	*Enterococcus faecium*	Meat	2,790,259	64	38	279,362	197,862	6	60
41	JAMXGL000000000	*Enterococcus faecium*	Meat	2,582,759	50	38	284,042	176,213	6	93
99	JAMXGH000000000	*Enterococcus faecalis*	Meat	2,800,179	24	37.6	1,019,444	414,094	2	84

### 3.4 Antimicrobial resistance determinants

Antimicrobial resistance genes conferring resistance to 9 different antimicrobial classes were detected among *E. faecium* (8/9; 89%), *E. faecalis* (8/9; 89%) and *E. lactis* (3/9; 33%) (Table 4). Genes encoding resistance to tetracyclines [*tet(M), tet(L), tet(S)*], lincosamides [*lsa(A), lsa(E), lnu(B)*], macrolides [*erm(B), erm(T), msr(C)*], aminoglycosides [*aac(6′)-Ii, aph(3′)-III, ant(6)-Ia, aac(6′)-aph(2″), str*], streptogramins [*lsa(A), lsa(E), msr(C)*], trimethoprim [*dfrG*] and amphenicol [*cat*] were detected among isolates (Table 4). Chromosomal point mutations were observed in *E. faecalis* genomes (2/24; 8.3%) in the quinolone resistance determinant regions (QRDRs) of the DNA gyrase (*gyrA* p.S83Y) and DNA topoisomerase IV genes (*parC p.S80I, parC p.S80R*). Similarly, *E. faecium* genomes (7/16; 44%) exhibited multiple point mutations in the *pbp5* gene which encodes ampicillin resistance (Table 4). The *ClpL* gene encoding resistance to heat was identified in 7/40 (18%) of *Enterococcus* spp. genomes. With a value of p of <0.001, chi-square test indicated a significant association between resistance phenotype and the presence of associated gene or genotype. Logistic regression also showed a three-fold increase in the odds of detecting associated genes for each unit increase in the different resistance phenotypes observed. Thus, a significant relationship between the type of resistance phenotype and genotype was observed.

**Table 4:** Characteristics of *Enterococcus* spp. resistant to ≥2 antimicrobial agents.

Isolate	ST	Source	Antibiotype	AMR Genes	PLASMID REPLICON	MGEs
*E. faecalis*	ST32 (*n* = 6)	Goat (*n* = 2), Sheep (*n* = 4)	ERY-TET-CIP	*aph(3′)-III, dfrG, erm(B), lsa(A), str, tet(L), tet(M)*	*rep7a, rep9a, repUS11, rep18b, repUS43, repUS58*	*ISS1N*
*E. faecalis*	ST1052 (*n* = 3)	Cattle (*n* = 2), Pig (*n* = 1)	ERY-TET	*aac(6′)-aph(2″), ant(6)-Ia, aph(3′)-III, dfrG, erm(B), lsa(A), tet(L), tet(M)*	*rep9b, repUS43*	*Tn6009, ISSsu5*
*E. faecalis*	ST1295* (*n* = 3)	Poultry (*n* = 3)	ERY-TET-CIP	*ant(6)-Ia, aph(3′)-III, erm(B), lsa(A), tet(L), tet(M)*	*rep1, rep9b, repUS43*	–
*E. faecalis*	ST1297* (*n* = 2)	Sheep (*n* = 2)	ERY-TET-CIP-CHL	*ant(6)-Ia, aph(3′)-III, erm(B), lsa(A), str, tet(L), tet(M)*	*rep7a, rep9b, repUS43*	*Tn917, ISEfm1*
*E. faecalis*	ST16 (*n* = 2)	Goat (*n* = 2)	ERY-TET	*aac(6′)-aph(2″), ant(6)-Ia, aph(3′)-III, dfrG, erm(B), lsa(A), tet(M)*	*repUS11, repUS43*	*Tn6009, ISLgar5, cn_12488_ISLgar5, ISSsu5*
*E. faecalis*	ST81 (*n* = 2)	Sheep (*n* = 2)	ERY-TET-CHL	*cat, dfrG, erm(B), lsa(A), str, tet(L), tet(M)*	*rep7a, rep9a, repUS43*	*ISEfa5*
*E. faecalis*	ST1306* (*n* = 1)	Meat (*n* = 1)	TET-CHL-LZD	*cat, lsa(A), str, tet(L), tet(M)*	*rep7a, rep9a, repUS43, repUS47*	*ISS1N*
*E. faecalis*	ST245 (*n* = 1)	Sheep (*n* = 1)	ERY-TET-CIP	*ant(6)-Ia, aph(3′)-III, erm(B), lsa(A), str, tet(L), tet(M), parC*	*rep6, rep7a, rep9b, repUS43*	*Tn917*
*E. faecalis*	ST300 (*n* = 1)	Poultry (*n* = 1)	ERY-TET-CIP	*ant(6)-Ia, aph(3′)-III, erm(B), lsa(A), tet(L), tet(M)*	*rep1, rep9b*	–
*E. faecalis*	ST4 (*n* = 1)	Poultry (*n* = 1)	ERY-TET-CHL	*aac(6′)-aph(2″), aph(3′)-III, cat, dfrG, erm(B), lnu(B), lsa(A), lsa(E), str, tet(L), tet(M)*	*rep7a, rep9a, rep9c, repUS11, repUS43*	–
*E. faecalis*	ST480 (*n* = 1)	Sheep (*n* = 1)	TET-CIP	*dfrG, lsa(A), tet(L), tet(M), gyrA, parC*	-	*Tn6009, ISEnfa364, ISEf1*
*E. faecalis*	ST86 (*n* = 1)	Goat (*n* = 1)	ERY-TET	*dfrG, erm(B), lsa(A), tet(L), tet(M)*	*rep9b, repUS43*	*ISEf1*
*E. faecium*	ST1442 (*n* = 2)	Sheep (*n* = 1), Meat (*n* = 1)	ERY-TET	*aac(6′)-Ii, msr(C), tet(L), tet(M), pbp5, ClpL*	*rep1, rep2, repUS15, repUS43*	*ISLgar5, ISEfa11, ISEfa10, IS256,ISS1N*
*E. faecium*	ST2269* (*n* = 2)	Meat (*n* = 1), Sheep (*n* = 1)	ERY-TET-CIP-CHL	*aac(6′)-Ii, cat, erm(B), msr(C), tet(L), tet(S)*	*rep1, rep14b, rep2, repUS15*	*ISS1N, ISEnfa4, ISEnfa1*
*E. faecium*	ST2237* (*n* = 1)	Meat (*n* = 1)	ERY-TET	*aac(6′)-Ii, msr(C)*	*rep1, rep2, repUS15*	*ISEfa10, ISEfa11, ISLgar5, ISEnfa1, ISEnfa4*
*E. faecium*	ST94 (*n* = 1)	Goat (*n* = 1)	ERY-TET	*aac(6′)-Ii, msr(C), tet(M), ClpL*	*rep1, repUS15, repUS43*	*Tn6009, ISEfa10, ISLgar5*
*E. lactis*	ST94 (*n* = 1)	Goat (*n* = 1)	ERY-TET	*aac(6′)-Ii, msr(C), ClpL*	*repUS15*	*ISEfa10*
*E. faecium*	ST12 (*n* = 1)	Goat (*n* = 1)	ERY-TET-CIP	*aac(6′)-Ii, erm(B), msr(C), tet(L), tet(M), pbp5*	*rep2, repUS15, repUS43*	*ISEnfa1, cn_5536_ISEnfa1, ISEfa4, ISEfa5*
*E. faecium*	ST2268* (*n* = 1)	Pig (*n* = 1)	ERY-TET-CHL	*aac(6′)-Ii, msr(C), ClpL*	-	*ISLgar5, ISEfm1*
*E. faecium*	ST1216 (*n* = 1)	Pig (*n* = 1)	ERY-TET	*aac(6′)-Ii, msr(C), pbp5*	*rep1, repUS15*	–
*E. faecium*	ST158 (*n* = 1)	Pig (*n* = 1)	ERY-TET	*aac(6′)-Ii, aac(6′)-aph(2″), ant(6)-Ia, aph(3′)-III, dfrG, erm(B), msr(C), tet(L), pbp5*	*rep2, rep22, rep9a, repUS15, repUS43*	*ISS1N, ISEfa4, ISSsu5, ISEnfa4*
*E. faecium*	ST1939 (*n* = 1)	Cattle (*n* = 1)	ERY-TET	*aac(6′)-Ii, msr(C), ClpL*	*rep1*	*ISS1N, ISEfm1, cn_2426_ISEfm1*
*E. faecium*	ST1980 (*n* = 1)	Sheep (*n* = 1)	ERY-TET	*aac(6′)-Ii, erm(B), msr(C), tet(L), tet(M)*	*rep1, rep2, repUS15, repUS43*	*Tn917, ISEnfa1, cn_5536_ISEnfa1, ISEfa10*
*E. faecium*	ST32 (*n* = 1)	Meat (*n* = 1)	ERY-CIP	*aac(6′)-Ii, msr(C), pbp5*	–	–
*E. faecium*	ST361 (*n* = 1)	Cattle (*n* = 1)	ERY-TET	*aac(6′)-Ii, msr(C), ClpL*	*repUS15*	*ISEfa10*
*E. faecium*	ST416 (*n* = 1)	Meat (*n* = 1)	ERY-TET-CHL	*aac(6′)-Ii, erm(T), msr(C), tet(L), tet(M), pbp5*	*repUS12, repUS43*	–

AMP, Ampicillin; VAN, Vancomycin; ERY, Erythromycin; TET, Tetracycline; CIP, Ciprofloxacin; CHL, Chloramphenicol; *novel sequence types. Enterococcus spp. are intrinsically resistant to aminoglycosides, sulphonamides and lincosamides, hence no tests were performed against these antimicrobial classes.

### 3.5 Virulence factors

Virulence genes were detected in all isolates sequenced. The genes present in *E. faecalis* isolates include biofilm formation-associated pili genes [*ebpA, ebpB, ebpC, SrtA*], adhesins [*ace, efaAfs, agg*], sex pheromones [*cad, camE, cCF10, cOB1*], cytolysins [*cylB, cylL, cylM*], hyaluronidase [*hylA, hylB*], gelatinase [*gelE*], oxidative stress resistance [*tpx*], quorum-sensing [*fsrB*] and anti-phagocytic activity [*ElrA*]. A narrower spectrum of virulence genes was detected in *E. faecium* and *E. lactis* genomes; *acm* and *efaAfm* all encode adhesins.

### 3.6 Mobile genetic elements

Majority of the isolates (39/40; 98%) sequenced possessed mobile genetic elements including integrative conjugative elements, plasmids, insertion sequences, unit and composite transposons (Table 4). A total of 16 different replicon plasmid sequences were identified in the study, including *repUS43* (*n* = 29), *rep7a* (*n* = 13), *rep1* (*n* = 12), *repUS15* (*n* = 12), *repUS11* (*n* = 11) and *rep9a* (*n* = 11).

AMR genes (28/40; 70%) were found to occur on the same contig as plasmid replicons. Of note, *tet(M)*, *tet(L)* and *erm(B)* genes were borne on *repUS43*, *rep9a* and *rep9b* plasmid replicons whereas *aph(3′)-III* and *ant(6)-Ia* genes were carried by *rep1* plasmid and *str* gene, *rep7a* plasmid (Table 5). Meanwhile, AMR genes encoding tetracycline, macrolide and aminoglycoside resistance were also identified to be associated with other mobile genetic elements such as transposons (*Tn917, cn_5536_ISEnfa1*), integrative conjugative element (*Tn6009*) and insertion sequences (*ISEnfa1, ISEfa10*) in 10/40 (25%) of the isolates (Table 5). The occurrence of virulence genes on plasmids or other mobile genetic elements was observed in 3/40 (8%) of isolates.

**Table 5:** Isolates with MGEs co-occurring with AMR genes on the same contig.

Isolate	Plasmid replicons	AMR genes	MGEs	AMR genes
*E. faecalis*				
18f	*repUS43*	*tet(M), lsa(A), tet(L)*	*–*	*–*
32f	*repUS43*	*tet(M), lsa(A), tet(L)*	*–*	*–*
38f	*repUS43*	*tet(M), lsa(A), tet(L)*	*Tn6009*	*tet(M), Lsa(A), tet(L)*
47f	*repUS43*	*tet(M), tet(L), aph(3′)-III*	*Tn917*	*tet(M), tet(L), aph(3′)-III*
	*rep9b*	*tet(M), tet(L), aph(3′)-III*		
	*rep7a*	*str*		
55f	*repUS43*	*tet(M)*	*Tn6009*	*tet(M)*
65	*repUS43*	*dfrG, tet(M), tet(L), erm(B)*	*–*	*–*
	*rep9b*	*dfrG, tet(M), tet(L), erm(B)*		
65f	*repUS43*	*tet(M)*	*Tn6009*	*tet(M)*
70	*repUS43*	*tet(M), tet(L)*	*–*	*–*
	*rep9a*	*tet(M), tet(L)*		
	*rep7a*	*str*		
71	*repUS43*	*tet(M), tet(L)*	*–*	*–*
	*rep9a*	*tet(M), tet(L)*		
	*rep7a*	*str*		
72f	*repUS43*	*tet(M), tet(L)*	*–*	*–*
	*rep9a*	*tet(M), tet(L)*		
	*rep7a*	*str*		
73	*repUS43*	*tet(M), tet(L), aph(3′)-III*	*Tn917*	*tet(M), tet(L), aph(3′)-III*
	*rep9a*	*tet(M), tet(L), aph(3′)-III*		
	*rep7a*	*str*		
74	*repUS43*	*tet(M), tet(L)*	*–*	*–*
	*rep9b*	*tet(M), tet(L)*		
	*rep7a*	*str*		
75	*repUS43*	*tet(M), tet(L)*	*Tn917*	*erm(B), aph(3′)-lll*
	*rep7a*	*str*		
	*rep9b*	*erm(B), aph(3′)-lll*		
76f	*repUS43*	*tet(M), tet(L), cat*	*–*	*–*
	*rep9a*	*tet(M), tet(L), cat*		
	*rep7a*	*str*		
78 m	*repUS43*	*tet(M), tet(L), cat*	*–*	*–*
	*rep9a*	*tet(M), tet(L), cat*		
	*rep7a*	*str*		
81	*repUS43*	*tet(M), tet(L)*	*–*	*–*
	*rep9a*	*tet(M), tet(L)*		
	*rep7a*	*str*		
85	*repUS43*	*tet(M)*	*Tn6009*	*tet(L)*
88 l	*repUS43*	*tet(M), tet(L)*	*–*	*–*
	*rep9b*	*tet(M), tet(L)*		
	*rep1*	*ant(6)-Ia, aph(3′)-lll*		
89	*rep9b*	*tet(M), tet(L)*	–	–
	*rep1*	*ant(6)-Ia, aph(3′)-lll*		
90	*repUS43*	*tet(M), tet(L), cat*	–	–
	*rep9a*	*tet(M), tet(L), cat*		
	*rep7a*	*str*		
91	*repUS43*	*tet(M), tet(L)*	–	–
	*rep9b*	*tet(M), tet(L)*		
	*rep1*	*ant(6)-Ia, aph(3′)-lll*		
99	*repUS43*	*tet(M), tet(L)*	*–*	*–*
	*rep7a*	*str*		
	*rep9a*	*tet(M), tet(L)*		
	*repUS47*	*cat*		
*E. faecium*				
41	*repUS43*	*tet(M), tet(L)*	*–*	*–*
	*repUS12*	*tet(M), tet(L)*		
67	*repUS43*	*tet(M), tet(L), erm(B)*	*ISEnfa1*	*tet(M), tet(L), erm(B)*
			*cn_5536_ISEnfa1*	*tet(M), tet(L), erm(B)*
67 M	*repUS43*	*tet(M)*	*Tn6009*	*tet(M)*
78	*repUS43*	*tet(M), erm(B)*	*Tn917*	*tet(M), tet(L), erm(B)*
			*ISEnfa1*	*tet(M), tet(L), erm(B)*
			*cn_5536_ISEnfa1*	*tet(M), tet(L), erm(B)*
79f	*repUS43*	*tet(M), tet(L)*	–	–
	*rep2*	*tet(M), tet(L)*		
82	*repUS43*	*tet(M), tet(L)*	–	–
	*rep7a*	*str*		
	*rep9a*	*tet(M), tet(L)*		

### 3.7 Multi-locus sequence typing

Eighteen *E. faecalis* isolates belong to ST32 (*n* = 6), ST1052 (*n* = 3), ST16 (*n* = 2), ST81 (*n* = 2), including two novel sequence types ST1295 (*n* = 3) and ST1297 (*n* = 2; Table 4). The remaining isolates had diverse sequence types (Table 4). *E. faecium* isolates belong to ST2269 (*n* = 2), ST1442 (*n* = 2), ST94 (*n* = 1), ST12 (*n* = 1), ST1216 (*n* = 1), ST158 (*n* = 1), ST1939 (*n* = 1), ST1980 (*n* = 1), ST32 (*n* = 1), ST361 (*n* = 1), ST2268 (*n* = 1), ST2237 (*n* = 1) and ST416 (*n* = 1). *E. lactis* ST94 (*n* = 1) was detected. *E. faecium* clones (ST1442 and ST2269) were identified in both meat and livestock samples. *E. faecium* isolates (2/15) identified belong to ST32 and ST416, which are part of the CC17 clonal complex known for causing hospital-acquired infections globally.

### 3.8 Phylogenetic analysis

**Figure 1:**
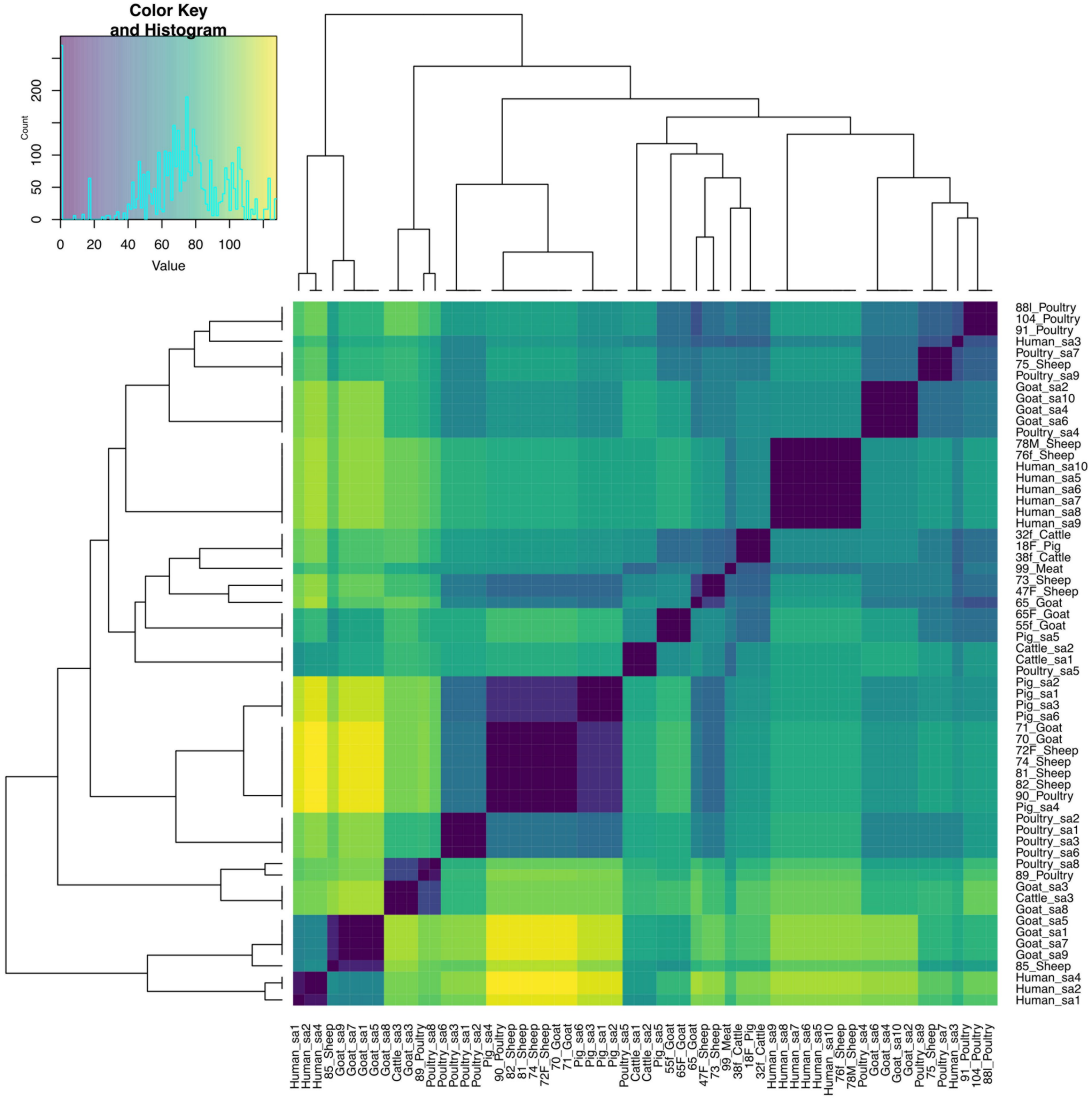
Heatmap and dendrogram showing pairwise SNP distance matrix and relationship among *E. faecalis* (*n* = 24) isolates in this study and other South African *E. faecalis* (*n* = 38) genomes downloaded from BV-BRC, labeled with the suffix “sa.” Isolates in this study included: Cattle = 2; Goat = 5; Meat = 1; Pig = 1; Poultry = 5 and Sheep = 10. The diagram shows the genetic variation between the isolates in the study. The pairwise distance matrix of SNPs showed close genetic relatedness between majority of the *E. faecalis* isolates from livestock in this study, with SNP difference below 10. Complete matrix data with pairwise distances recorded has been included in Supplementary material. Heatmap and dendrogram were created with gplots (v.3.1.3) and dendextend (v.3.1.3) packages, respectively, in R.

**Figure 2:**
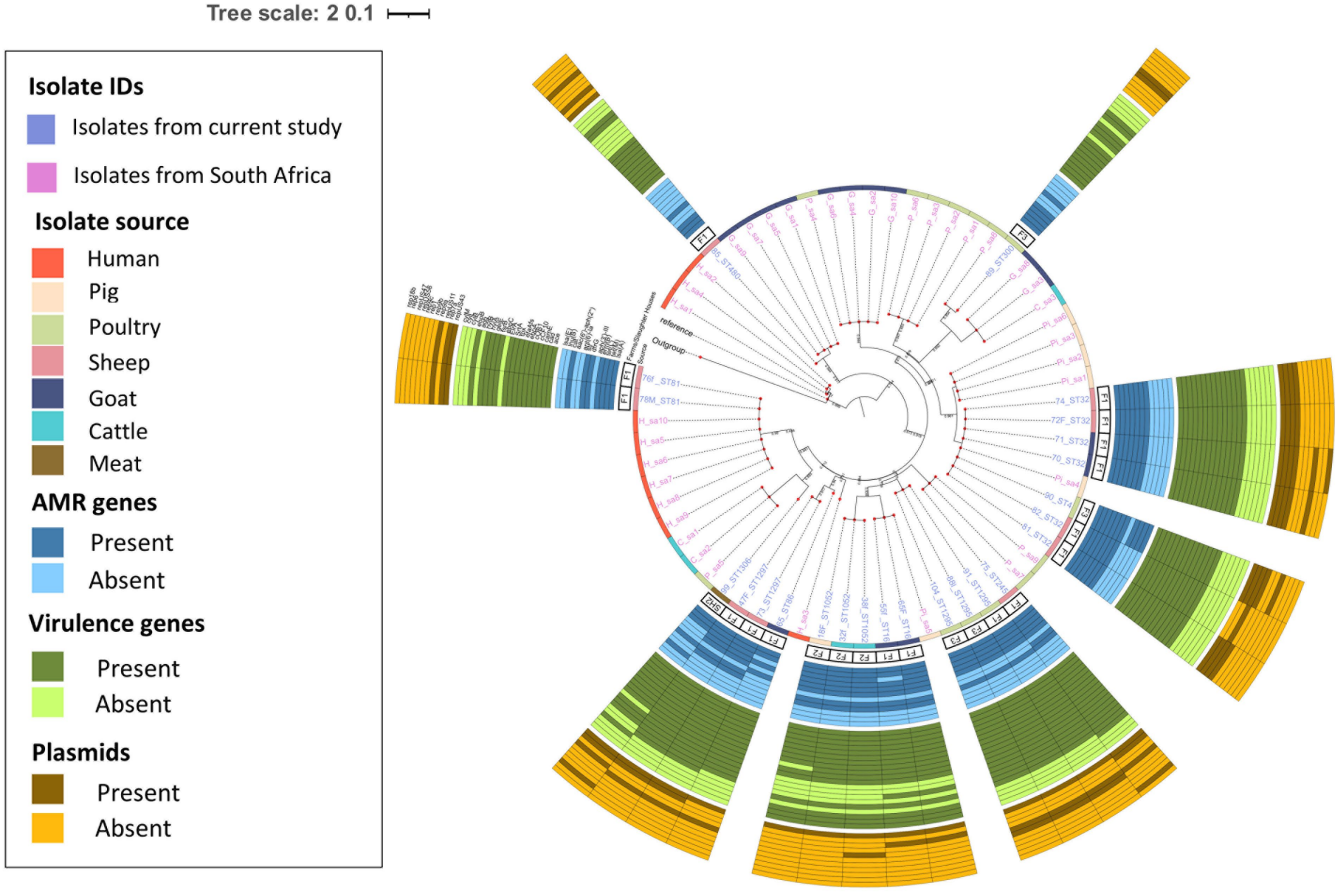
Population structure of *Enterococcus faecalis* isolates. Phylogeny of 24 study isolates and 38 isolates from South Africa downloaded from BV-BRC. The genomes were compared using *E. faecalis* V583 strain (226185.9) as reference. SNP-based maximum likelihood tree was constructed using CSI Phylogeny and visualized in iTOL. This phylogenetic tree shows the evolutionary relationship between *E. faecalis* isolates in this study and other *E. faecalis* genomes from South Africa. Clustering between *E. faecalis* isolates from livestock was mostly observed among isolates in this study and other genomes from South Africa. For each isolate in this study, the source, farm (F)/slaughter house (SH), the sequence types, AMR, virulence genes and plasmids distribution are shown.

The pairwise distance matrix of SNPs showed close genetic relatedness between majority of the *E. faecalis* isolates from livestock in this study (Figure 1). Clustering of *E. faecalis* isolates was mostly observed between isolates from the same farm (Figure 2). *E. faecalis* isolates recovered in this study also showed close genetic relatedness with the South African livestock genomes irrespective of the livestock type (Figures 1, 2). Two isolates from sheep (76F and 78 M) in this study were found clustering together with South African human clinical isolates, with a pairwise SNP difference below 10 (Figures 1, 2). Similar patterns in the distribution of plasmid replicons, AMR and virulence genes were seen among *E. faecalis* genomes occurring in the same clade (Figure 2).

**Figure 3:**
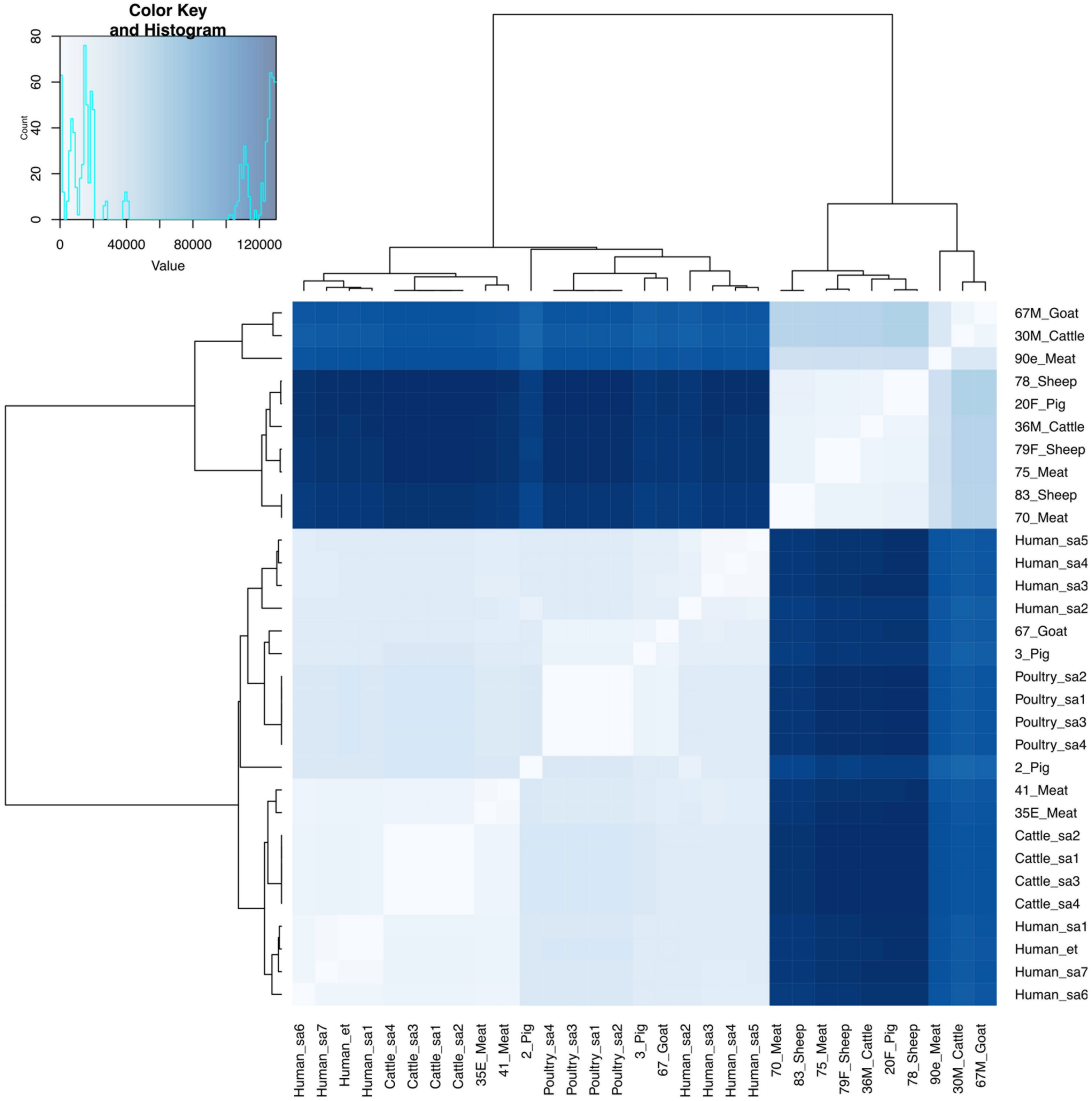
Heatmap and dendrogram showing pairwise SNP distance matrix and relationship among *E. faecium* (*n* = 15) isolates in this study and other *E. faecium* (*n* = 16) genomes downloaded from BV-BRC, labeled with the suffix “sa” and “et”. Isolates in this study included: Cattle = 2; Goat = 2; Meat = 5; Pig = 3 and Sheep =3. The diagram shows the genetic variation between the isolates in the study. The pairwise distance matrix of SNPs showed significant genetic diversity between *E. faecium* isolates, with SNP difference greater than 10. Complete matrix data with pairwise distances recorded has been included in Supplementary material. Heatmap and dendrogram were created with gplots (v.3.1.3) and dendextend (v.3.1.3) packages, respectively, in R.

**Figure 4:**
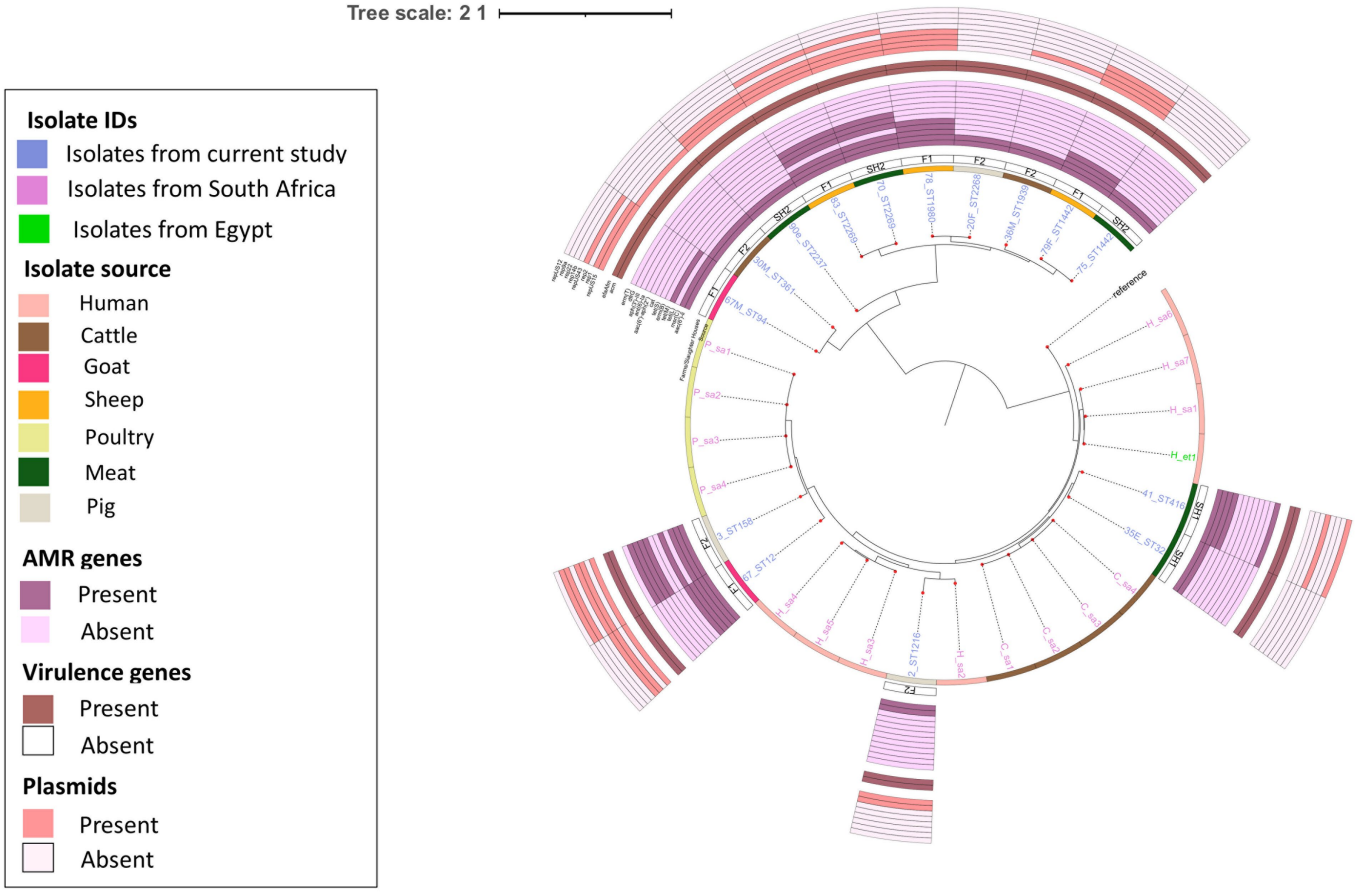
Population structure of *E. faecium* isolates. Phylogeny of 15 study isolates and 16 isolates from South Africa downloaded from BV-BRC. The genomes were compared using *E. faecium* SRR24 (NZ CP038996.1) as a reference. SNP-based maximum likelihood tree was constructed using CSI Phylogeny and visualized in iTOL. This phylogenetic tree shows the evolutionary relationship between *E. faecalis* isolates in this study and other *E. faecalis* genomes from South Africa. Limited genetic similarity between *E. faecium* isolates from meat and livestock was observed. For each isolate in this study, the source, farm (F)/slaughter house (SH), the sequence types, AMR, virulence genes and plasmids distribution are shown.

*E. faecium* isolates from livestock sources exhibited greater genetic diversity compared to those from South Africa (Figure 3). *E. faecium* isolates from meat and livestock were mostly genetically divergent (Figure 4). However, one *E. faecium* isolate from meat (isolate 70) showed close genetic relatedness to an isolate from sheep (isolate 83) in the livestock group, with a SNP difference below 10 (Figures 3, 4).

## 4 Discussion

This study revealed a substantial diversity among the isolates collected from both meat and livestock samples. While *E. faecalis* and *E. faecium* were the most prevalent species detected among the samples, no statistically significant differences were noted in the distribution of *Enterococcus* spp. This lack of differentiation among the *Enterococcus* spp. recovered from the various sources, suggests that these isolates may not exhibit strong niche-specific adaptions based on the particular source or animal host, contrary to reports elsewhere (Zaheer et al., 2020). The variation in the distribution of *Enterococcus* spp. among livestock across different geographic regions can be attributed to differences in dietary patterns, which impact the composition of gut commensal bacteria (Klibi et al., 2013; Ngbede et al., 2017). Similar to our findings, other studies (Golob et al., 2019; Habib et al., 2022) have also reported consistent contamination of retail meat by *Enterococcus* spp. and this has been linked to fecal contamination. Furthermore, the presence of antimicrobial resistance genes in these Enterococci isolates, including those related to clinically important antimicrobials like aminoglycosides, streptogramin A, macrolides, and tetracycline, suggests a narrowing of available treatment options (beta-lactam monotherapy or beta-lactam in combination with an aminoglycoside or glycopeptide) for opportunistic Enterococcus infections including those caused by vancomycin resistant strains (Arias et al., 2010; O’Driscoll and Crank, 2015). Among the isolates resistant to two or more antimicrobial agents, resistance to erythromycin, tetracycline, ciprofloxacin and chloramphenicol was common. The incidence of erythromycin and tetracycline resistance in *Enterococcus* spp. has been reported in other studies in Africa, and has been associated with the extensive use and misuse of these antimicrobials in animal production (Iweriebor et al., 2015; Ngbede et al., 2017; Molechan et al., 2019). The increased levels of resistance recorded against tetracycline could be attributed to the use of tetracycline as recorded in the livestock farms sampled for this study. Nonetheless, erythromycin resistance might also be due to cross resistance associated with another macrolide, tylosin documented to be used among livestock in Ghana (Jackson et al., 2004; Akansale et al., 2019). Resistance to erythromycin among *Enterococcus* spp. was found to be mediated by erythromycin ribosomal methylation (*erm*) genes which encode for the modification of ribosomal target through methylation and *msr(C)* genes which mediate the extrusion of the antibiotic (Munita and Arias, 2016). Moreover, the detection of *tet(L), tet(S) and tet(M)* genes indicate that tetracycline resistance was due to ribosomal target protection and efflux-mediated mechanisms (Munita and Arias, 2016). Chloramphenicol resistance was mediated by the chloramphenicol acetyltransferase (*cat*) gene responsible for inactivation of chloramphenicol antibiotic, while ciprofloxacin resistance was encoded by *gyrA* and *parC* mutations in the subunits of the DNA gyrase and topoisomerase IV enzymes (Munita and Arias, 2016).

Furthermore, the presence of *ClpL* gene suggests exposure of these *Enterococcus* spp. to heat and other adverse environmental conditions. These environmental stress factors have been demonstrated to select for resistant bacteria and influence susceptibility to a range of antibiotics (Huang et al., 2021). The finding of low resistance to ampicillin, linezolid and vancomycin is in line with other reports (Mannu et al., 2003; Bortolaia et al., 2016). Although linezolid resistance is considered uncommon due to its limited use, findings show that the use of other antimicrobials such as macrolides may select for resistance against linezolid (Tyson et al., 2018).

All three livestock farms sampled in this study used antibiotics including tetracycline, penicillin and colistin, similar to other studies in the country where antibiotic use has been described as inappropriate and unregulated (Donkor et al., 2012; Osei Sekyere, 2014; Boamah et al., 2016; Nkansa, 2020). Enterococci notably acquire AMR genes through plasmids and transposons, chromosomal exchange or mutation (Iweriebor et al., 2015; Torres et al., 2018). Mobile genetic elements including integrative conjugative elements, plasmid replicons, insertion sequences and transposons were identified among the isolates confirming the potential of these isolates to acquire and transfer AMR determinants between like-species and other pathogens. Co-occurrence between plasmid replicons (*rep1, rep2, repUS43, repUS47, rep9a, rep9b*) and AMR genes on the same contig was observed in 70% (28/40) of the isolates. Tetracycline resistance genes [*tet(M),tet(L)*] and macrolide resistance gene [*erm(B)*] more frequently co-existed with *repUS43, rep9a* and *rep9b* plasmid replicons. The association between *tet(M)* and *tet(L)* and r*epUS43* and *rep9b* was also reported in a similar study (Fatoba et al., 2022). Aminoglycoside resistance genes [*aph(3′)-III*, *ant(6)-Ia*] tended to be more frequently associated with *rep1* plasmid, while another aminoglycoside resistance gene *(str)* often occurred on the same contig with *rep7a* plasmid replicon. Other mobile genetic elements commonly associated with AMR genes encoding resistance to tetracycline, macrolides and aminoglycosides included transposons (*Tn917, cn_5536_ISEnfa1*), integrative conjugative element (*Tn6009*) and insertion sequences (*ISEnfa1, ISEfa10*). Macrolide resistance gene [*erm(B)*] was frequently associated with *Tn917* consistent with reports elsewhere (Torres et al., 2018). The abundance of plasmid replicon genes and other mobile genetic elements detected in this study confirms the plasticity of the genomes of *Enterococcus* spp. and supports the potential for horizontal dissemination of the AMR genes (Fatoba et al., 2022). The dissemination of these genes is intensified by clonal expansion which results when horizontally transferred AMR genes become chromosomally integrated (Waddington et al., 2022).

MSLT analysis revealed diverse clones including novel sequence types for both *E. faecium* and *E. faecalis* isolates. Novel sequence types for both *E. faecalis* and *E. faecium* harbored genes for virulence and resistance with demonstrated phenotypic resistance. The most common African clone, *E. faecalis* ST16, (Jolley et al., 2018) was found among the isolates (*n* = 2) in this study. The other clones (ST4, ST16, ST32, ST300) detected, have also been reported in Nigeria and South Africa from similar sources (Jolley et al., 2018). Clonal relationship was observed in four *E. faecium* isolates [ST1442 (*n* = 2), ST2269 (*n* = 2)] from meat and livestock samples. *E. faecium* ST32 and ST416 recovered from meat clustered with sequence types belonging to the hospital-adapted CC17, known to cause hospital-acquired and clinical infections worldwide (Leavis et al., 2006). Majority of isolates belonging to CC17 are characterized by resistance to quinolones and ampicillin, and the presence of enterococcal surface protein (Leavis et al., 2006; Top et al., 2008). *E. faecium* isolates (ST32 and ST416) showed resistance to ciprofloxacin but were both susceptible to ampicillin phenotypically, although mutations in the *pbp5* gene was detected for both isolates.

The pathogenicity of bacterial species requires not only antibiotic resistance but also the possession of specific virulence factors. The adherence of bacterial cells to host tissues is a critical stage in the development of infection or forming biofilms (Stępień-Pyśniak et al., 2019). In this study, both *acm* and *efaAfm* virulence genes were detected in all *E. faecium* isolates. These genes have also been documented in clinically-derived *E. faecium* isolates in the United States and Malaysia (Nallapareddy et al., 2008; Soheili et al., 2014). Acm gene, known for encoding an adhesin responsible for *E. faecium* attachment to collagen, plays a significant role in the competitiveness of this species within clinical environments and is crucial for survival, colonization and infection (Nallapareddy et al., 2008). While the precise function of *efaAfm* is yet to be confirmed, it is hypothesized to be involved in cell wall adherence (Soheili et al., 2014; Stępień-Pyśniak et al., 2019). Consistent with other reports (Jahansepas et al., 2018; Farman et al., 2019; Trościańczyk et al., 2022), a broader spectrum of virulence genes was identified in *E. faecalis* than *E. faecium,* suggesting that *E. faecalis* is more virulent than *E. faecium*. Genes mediating adherence to collagen (*ace*), biotic and abiotic surfaces (*efaAfs*) and expression of pili on cell surface (*ebpA, ebpB, ebpC, SrtA*) were all identified in *E. faecalis* isolates, facilitating cell adhesion and biofilm formation. Genes encoding sex pheromones (*cad, camE, cCF10, cOB1*) identified in all *E. faecalis* isolates are noted for promoting biofilm formation and regulation by inducing conjugation between enterococcal cells and mediating the transfer of pheromone-responsive plasmids, which may contain virulence genes (Stępień-Pyśniak et al., 2019; Yoon et al., 2020; Trościańczyk et al., 2022). Furthermore, genes encoding cytolysin (*cylB, cylL, cylM*), gelatinase (*gelE*), and hyaluronidase (*hylA, hylB*), which are known for their ability to damage cells were detected in *E. faecalis* isolates (Golińska et al., 2013; Stępień-Pyśniak et al., 2019; Lang et al., 2020). Although studies suggest that hyaluronidase encoded by *hyl* is specific for *E. faecium* it has been reported in *E. faecalis* isolates in other studies (Golob et al., 2019; Kiruthiga et al., 2020). *ElrA* and *tpx* genes, which are associated with evading host’s immune defenses were also detected, possibly indicating an evolved mechanism in these *E. faecalis* isolates for immune system evasion. Similar virulence factors have been isolated from *E. faecalis* isolates from food, animal, and clinical sources in Africa, Europe and Asia (Iweriebor et al., 2015, 2016; Beshiru et al., 2017; Jahansepas et al., 2018; Farman et al., 2019; Stępień-Pyśniak et al., 2019; Yoon et al., 2020). The ST16, ST4 and ST480 *E. faecalis* identified in this study, have also been reported in cases of clinical infection in other geographical regions (Watanabe et al., 2009;Olsen et al., 2012; Poulsen et al., 2012; Farman et al., 2019) suggesting the widespread nature of these clones. Similar to findings in this study, *E. faecalis* ST480 identified in Saudi Arabia carried virulence genes encoding sex pheromones [*cOB1*], biofillm formation-associated pili genes [*ebpA, ebpB, SrtA*] and adhesins [*ace, efaAfs*], and were resistant to erythromycin, ciprofloxacin and tetracycline (Farman et al., 2019). Similarly, *E. faecalis* ST16 isolates were mostly resistant to erythromycin, ciprofloxacin and tetracycline (Farman et al., 2019), and harbored virulence genes encoding cytolysin [*cylB, cylL, cylM*] and adhesins [*efaAfs, ace*] (Olsen et al., 2012; Poulsen et al., 2012; Farman et al., 2019). On the other hand, *E. faecalis* ST4 isolate in this study was resistant to erythromycin, tetracycline and chloramphenicol, and had similar virulence genes encoding biofilm formation [*gelE*] and adhesins [*efaAfs*] as *E. faecalis* ST4 isolated in Japan (Watanabe et al., 2009).

Phylogenetic analysis revealed close genetic relatedness among *E. faecalis* isolates from livestock sources in this present study and other livestock isolates from South Africa. In contrast to Mbanga et al. (2021), close genetic relatedness was observed among two sheep enterococci isolates and human clinical isolates obtained from South Africa. The enterococci isolates from sheep (76F and 78 M) harbored resistance genes for amphenicols, macrolides, tetracyclines, aminoglycosides, streptogramins and lincosamides, with multiple virulence factors as well. The identified evolutionary relationship between *E. faecalis* isolates from livestock in this study and those from human clinical sources in South Africa suggests *E. faecalis* originating from animals could potentially serve as a reservoir for human-associated *Enterococcus* species. Meanwhile, *E. faecium* isolates from livestock and meat sources in this study showed significant genetic differences. Livestock-derived *E. faecium* genomes obtained from South Africa showed some genetic similarity, albeit genetically diverse from isolates in this study. The alignment of *E. faecium* isolates to geographically distinct clusters is similar to a report by (Freitas et al., 2020), and indicates that these *E. faecium* populations evolved independently in distinct host environments and geographic regions. Similarly, the rare occurrence of genetic similarity between meat- and livestock-derived *Enterococcus* spp. may result from slaughter houses sourcing livestock from diverse farms in different geographical settings.

To the best of our knowledge, this is the first study reporting on WGS of *Enterococcus* spp. in Ghana. The study demonstrated the presence of AMR *Enterococcus* spp. from livestock and raw meat samples in Ghana, and highlighted the potential of these bacteria to serve as reservoirs for resistant genes and further transmission to other pathogenic bacteria. The study also described the mechanisms of resistance exhibited by *Enterococcus* spp. against important antimicrobial agents. Genomic analysis also detected hospital-acquired strains and other clinically relevant *Enterococcus* spp. Phylogenetic analysis revealed evolutionary relationship among various livestock and meat isolates as well as demonstrated the potential of these isolates evolving to cause infections in humans. The use of WGS in this study has provided granular data for AMR surveillance from a one-health perspective to help consolidate efforts to strengthen public health surveillance systems in Ghana.

## Data availability statement

The datasets presented in this study can be found in online repositories. The names of the repository/repositories and accession number(s) can be found in the article/Supplementary material.

## Ethics statement

The animal study was approved by Ethical clearance for this study was obtained from the institutional review board (IRB) and Animal Care and Use Committee at Noguchi Memorial Institute for Medical Research (FWA00001824). The study was conducted in accordance with the local legislation and institutional requirements.

## Author contributions

GA: Data curation, Formal analysis, Investigation, Methodology, Software, Writing – original draft, Writing – review & editing. ED: Data curation, Formal analysis, Investigation, Methodology, Software, Writing – review & editing, Conceptualization, Funding acquisition, Resources, Supervision, Validation, Visualization. CO-N: Data curation, Formal analysis, Investigation, Methodology, Software, Writing – review & editing. FAO: Methodology, Writing – review & editing. QM: Methodology, Writing – review & editing. PN: Funding acquisition, Writing – review & editing. BA: Writing – review & editing, Methodology. RH: Writing – review & editing, Funding acquisition, Project administration, Resources, Validation, Visualization. BE: Funding acquisition, Project administration, Resources, Validation, Visualization, Writing – review & editing, Conceptualization, Data curation, Formal analysis, Investigation, Methodology, Software, Supervision, Writing – original draft.

## Footnotes

^1^ http://www.genomicepidemiology.org/

^2^ https://www.bv-brc.org/
