# Supplementary material for: Enterococcus species: insights into antimicrobial resistance and whole-genome features of isolates recovered from livestock and raw meat in Ghana
Source: Front Microbiol. 2023 Dec 5;14:1254896. doi: 10.3389/fmicb.2023.1254896 (PMC10773571; doi:10.3389/fmicb.2023.1254896)
Supplement: Supplementary file 1 [file Data_Sheet_1.zip › Genome accession for downloaded Enterococcus genomes.docx]

Table 1: Genome accession of E. faecalis genomes downloaded from the Bacterial and Viral Bioinformatics Resource Center (BV-BRC)

| Genome Name* | Genome Accession |
| --- | --- |
| Cattle_SA1 | JAHHES010000001 |
| Cattle_SA2 | JAHHEU010000001 |
| Cattle_SA3 | JAHHEV010000001 |
| Goat_SA1 | JAHHEY010000001 |
| Goat_SA2 | JAHHEX010000001 |
| Goat_SA3 | JAHHEW010000001 |
| Goat_SA4 | JAHHEX010000001 |
| Goat_SA5 | JAHHEY010000001 |
| Goat_SA6 | JAHHEX010000001 |
| Goat_SA7 | JAHHEY010000001 |
| Goat_SA8 | JAHHEW010000001 |
| Goat_SA9 | JAHHEY010000001 |
| Goat_SA10 | JAHHEX010000001 |
| Pig_SA1 | JAHHFH010000001 |
| Pig_SA2 | JAHHFH010000001 |
| Pig_SA3 | JAHHFH010000001 |
| Pig_SA4 | JAHHEQ010000001 |
| Pig_SA5 | JAHHER010000001 |
| Pig_SA6 | JAHHFH010000001 |
| Poultry_SA1 | JAHHFD010000001 |
| Poultry_SA2 | JAHHFD010000001 |
| Poultry_SA3 | JAHHFD010000001 |
| Poultry_SA4 | JAHHFB010000001 |
| Poultry_SA5 | JAHHFC010000001 |
| Poultry_SA6 | JAHHFD010000001 |
| Poultry_SA7 | JAHHFF010000001 |
| Poultry_SA8 | JAHHFE010000001 |
| Poultry_SA9 | JAHHFG010000001 |
| Human_SA1 | NXKG01000001 |
| Human_SA2 | NZ_PGCW00000000 |
| Human_SA3 | NZ_PGCX00000000 |
| Human_SA4 | NZ_PGCV00000000 |
| Human_SA5 | JADBPL010000001 |
| Human_SA6 | JADBPF010000001 |
| Human_SA7 | JADBPB010000001 |
| Human_SA8 | JADBPE010000001 |
| Human_SA9 | JADBPC010000001 |
| Human_SA10 | JADBOZ010000001 |

* Names refer to IDs as used in the phylogenetic analysis

Table 2: Genome accession of E. faecium genomes downloaded from the Bacterial and Viral Bioinformatics Resource Center (BV-BRC)

| Genome Name* | Genome Accession |
| --- | --- |
| Cattle_SA1 | JAHHFJ010000001 |
| Cattle_SA2 | JAHHFJ010000001 |
| Cattle_SA3 | JAHHFJ010000001 |
| Cattle_SA4 | JAHHFJ010000001 |
| Human_ET | CP064279 |
| Human_SA1 | NXIX01000001 |
| Human_SA2 | NZ_PGCS00000000 |
| Human_SA3 | NZ_PGCT00000000 |
| Human_SA4 | NZ_PGCU00000000 |
| Human_SA5 | NZ_PGCR00000000 |
| Human_SA6 | JADBPD010000001 |
| Human_SA7 | AHXO01000001 |
| Poultry_SA1 | JAHHFA010000001 |
| Poultry_SA2 | JAHHFA010000001 |
| Poultry_SA3 | JAHHFA010000001 |
| Poultry_SA4 | JAHHFA010000001 |

* Names refer to IDs as used in the phylogenetic analysis
